# Large-scale determination of absolute phosphorylation stoichiometries in human cells by motif-targeting quantitative proteomics

**DOI:** 10.1038/ncomms7622

**Published:** 2015-03-27

**Authors:** Chia-Feng Tsai, Yi-Ting Wang, Hsin-Yung Yen, Chih-Chiang Tsou, Wei-Chi Ku, Pei-Yi Lin, Hsuan-Yu Chen, Alexey I. Nesvizhskii, Yasushi Ishihama, Yu-Ju Chen

**Affiliations:** 1 Department of Chemistry, National Taiwan University, Taipei 10617, Taiwan; 2 Institute of Chemistry, Academia Sinica, 128 Academia Road, Section 2, Taipei 11529, Taiwan; 3 Chemical Biology and Molecular Biophysics Program, Taiwan International Graduate Program, Academia Sinica, 128 Academia Road, Section 2, Taipei 11529, Taiwan; 4 Institute of Biochemical Sciences, National Taiwan University, Taipei 10617, Taiwan; 5 Genomics Research Center, Academia Sinica, 128 Academia Road, Section 2, Taipei 11529, Taiwan; 6 Department of Computational Medicine and Bioinformatics and Department of Pathology, University of Michigan Medical School, Ann Arbor, Michigan 48109, USA; 7 School of Medicine, Fu Jen Catholic University, New Taipei City 24257, Taiwan; 8 Institute of Statistical Science, Academia Sinica, 128 Academia Road, Section 2, Taipei 11529, Taiwan; 9 Graduate School of Pharmaceutical Sciences, Kyoto University, Kyoto 606-8501, Japan

## Abstract

Our ability to model the dynamics of signal transduction networks will depend on accurate methods to quantify levels of protein phosphorylation on a global scale. Here we describe a motif-targeting quantitation method for phosphorylation stoichiometry typing. Proteome-wide phosphorylation stoichiometry can be obtained by a simple phosphoproteomic workflow integrating dephosphorylation and isotope tagging with enzymatic kinase reaction. Proof-of-concept experiments using CK2-, MAPK- and EGFR-targeting assays in lung cancer cells demonstrate the advantage of kinase-targeted complexity reduction, resulting in deeper phosphoproteome quantification. We measure the phosphorylation stoichiometry of >1,000 phosphorylation sites including 366 low-abundance tyrosine phosphorylation sites, with high reproducibility and using small sample sizes. Comparing drug-resistant and sensitive lung cancer cells, we reveal that post-translational phosphorylation changes are significantly more dramatic than those at the protein and messenger RNA levels, and suggest potential drug targets within the kinase–substrate network associated with acquired drug resistance.

## Abstract (web summary)

Measuring phosphorylation stoichiometry on a proteomic scale remains a challenge. Tsai *et al*. develop a technique to measure the basal level of phosphorylation stoichiometry in a single human phosphoproteome and identify molecular changes associated with gefitinib resistance in lung cancer cells.

## Graphical abstract

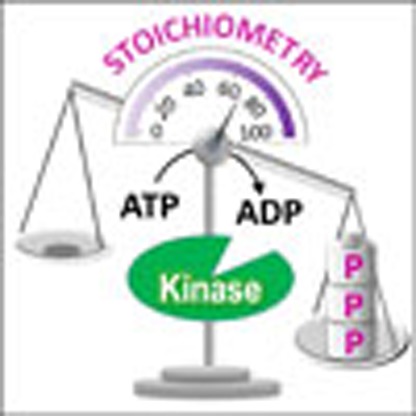

Deregulated signalling through protein phosphorylation is closely linked to pathogenesis of human cancer^1^. Measurement of the phosphorylation event is often used as an indicator of signalling pathway activation. Despite high sensitivity, the applicability of conventional immunoassays for phosphorylation sites is restricted owing to the limited availability of phosphosite-specific antibodies^2^,^3^,^4^. Advances in mass spectrometry-based quantitation approaches have revealed the system view of the phosphorylation-mediated network based on site-specific phosphorylation. However, one limitation of most quantitative phosphoproteomic studies is their inability to differentiate changes in phosphorylation stoichiometry from changes in protein abundance. The term ‘phosphorylation stoichiometry’ at a particular site is defined as the ratio of the total amount of protein phosphorylated on the site to the total amount of protein^2^,^3^,^4^. Only direct measurement of the phosphorylation stoichiometry can unambiguously reveal whether the signal-induced alteration is regulated by upstream kinase/phosphatase activity or by transcriptional regulation to modify protein abundance. Thus, measuring phosphorylation stoichiometry enables an in-depth understanding of the regulation mechanisms of cellular signalling networks^5^.

Pioneering work on large-scale analyses of protein phosphorylation stoichiometry was reported by Olsen *et al*.^6^ The stoichiometry of 5,000 phosphorylation sites during mitosis was calculated by three ratios of ion signals of phosphopeptides, unmodified counterparts and their corresponding proteins obtained by extensive fractionation and quantification of the phosphoproteome and proteome. Despite its success, the requirement of a minimum of three ratios under different cell statuses limited its application in calculating the site stoichiometry for a single cellular condition. To calculate the phosphorylation stoichiometry, the ratios of extracted ion signals on the modified peptide, unmodified peptides and corresponding protein ratios between two biological states have to be obtained (Supplementary Note 1).^6^

Wu *et al*.^7^ reported another approach applicable to single cell status, in which phosphatase treatment and isotopic labelling were integrated with hydrophilic interaction chromatography fractionation to identify as many dephosphorylated peptides as possible that match previously reported phosphopeptide sequences. This approach was applied to the relatively less complex yeast proteome to obtain the basal distribution of site stoichiometry for >5,000 phosphorylation events from phosphatase-treated and mock-treated samples from wild-type yeast. Due to the significantly greater complexity of the human proteome, until now, there has been no well-established approach for phosphorylation site stoichiometry measurement for a single cellular condition.

To overcome the current bottleneck in accessing the stoichiometry of single-state human phosphoproteomes, we have developed a motif-targeting quantitative proteomic approach. By using kinase reactions and immobilized metal ion affinity chromatography (IMAC), this approach can enrich these initially unphosphorylated peptides, that is, the counterpart of the phosphorylated form, which effectively reduces complexity of kinase–substrate peptides prior to stoichiometry measurement by liquid chromatography-tandem mass spectrometry (LC-MS/MS) analysis. To our knowledge, this method is the first large-scale approach to provide system-wide phosphorylation stoichiometry of a single-state human proteome. In addition to the demonstrated sensitivity, reliability and accuracy, this assay offers the advantage of kinase-targeted complexity reduction for deeper phosphoproteome analysis; hundreds of tyrosine phosphopeptides could be detected from only 50 μg cell lysates. The practicality of this approach was demonstrated on the quantitative measurement of drug-resistant and sensitive lung cancer cell lines; this motif-targeting approach differentiates drug resistance-associated changes in phosphorylation stoichiometry from those at the protein as well as messenger RNA (mRNA) levels in lung cancer cells, which also suggested potential druggable target proteins in the EGFR- and CK2-centred kinase–substrate network.

## Results

### Workflow of motif-targeting quantitation approach

**Figure 1:**
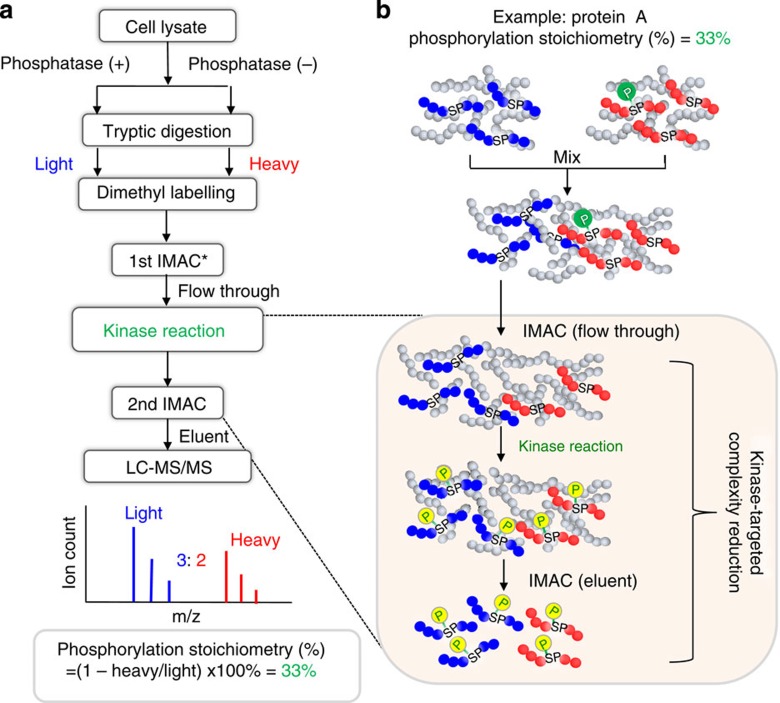
The basic principle of the motif-targeting quantitative proteomic approach. (**a**) Two identical aliquots of tryptic peptides are either mock or phosphatase treated followed by isotopic tagging. Then, the mixing fraction is purified by IMAC. In the flow through of IMAC purification (**b**), the dephosphorylated peptides from phosphatase-treated aliquot (blue, light isotope labelled) will represent the total peptides, while the unphosphorylated counterparts in the untreated aliquot (red, heavy isotope labelled) will represent the fraction of initial unphosphorylated amount. The mixture of dephosphorylated and unphosphorylated peptides in the flow through is subjected to phosphorylation via a kinase reaction. The motif-targeting phosphopeptides are purified by IMAC. The ratio of heavy/light will represent the fraction of initially unphosphorylated amount and the phosphorylation stoichiometry can be calculated by the formula shown in **a**. *: In this IMAC step, the phosphopeptides identified from the IMAC eluent can be used to derive the phosphorylation sequence motif and potential kinase for subsequent kinase reaction.

In this study, we developed a motif-targeting quantitative proteomic approach for the large-scale measurement of absolute site stoichiometry in the human phosphoproteome. This workflow integrates dephosphorylation and isotope tagging with an enzymatic kinase reaction. As shown in Fig. 1, two identical aliquots of tryptic peptides from the lysate are either mock- or phosphatase-treated followed by isotopic tagging with dimethyl labelling. The two fractions are then recombined and purified by IMAC. In the flow through of IMAC purification, the dephosphorylated peptides (including initially unphosphorylated peptides) from phosphatase-treated aliquot will represent the total peptides, while the unphosphorylated counterparts in the untreated aliquot will represent the fraction of initially unphosphorylated amount. The eluted phosphopeptides are analyzed by LC-MS/MS. The eluent of IMAC (mainly heavy labelled phosphopeptides) in this step allows identification of the phosphopeptides and Motif-X analysis^8^ to facilitate selection of a kinase relevant to the cell type (such as ERK2 and CK2 used in this study) for subsequent kinase reaction. For kinase selection, it is also noted that any kinase of interest, such as EGFR as demonstrated in this study, can be chosen independent of Motif-X analysis. In addition, the IMAC eluent also allow estimation of phosphatase treatment efficiency, which is based on the ratio between the light isotope signal intensity for ‘surviving’ phosphopeptides from the phosphatase-treated sample and heavy isotope signal intensity from the mock-treated initially phosphorylated peptides.

In the last step, the dephosphorylated peptides and unphosphorylated counterparts in the flow through of IMAC are subjected to phosphorylation via a kinase reaction (Fig. 1b). In most previous reports^7^,^9^, the phosphorylation site stoichiometry was determined by unphosphorylated and dephosphorylated peptides from phosphatase-treated and mock-treated sample. However, due to the significantly greater complexity of human proteome, the signal of initially unphosphorylated and dephosphorylated peptides with light labelling (phosphatase treated) and initially unphosphorylated peptides with heavy labelling (mock treated) would be suppressed by other kinds of mostly unmodified peptides. In addition, the limiting MS/MS sampling speed would also affect the identification coverage of these unphosphorylated and dephosphorylated peptides in complex human proteome. Therefore, it will be difficult to fully identify all the heavy/light pair for ratio determination. On the other hand, the second IMAC following kinase treatment can enrich these unphosphorylated and dephosphorylated peptides, which effectively reduce complexity of kinase–substrate peptides prior to stoichiometry measurement by LC-MS/MS analysis. The specific motif recognition by the selected kinase allows selective phosphorylation of target peptides of interest, for example, the low-abundance tyrosine phosphorylated peptides. By combining the second IMAC enrichment, the complexity of the first IMAC flow through is significantly reduced, while the heavy/light ratio of the target peptides phosphorylated from the unphosphorylated and dephosphorylated forms represent the ratio of the initial unphosphorylated amount to the total peptide amount (Fig. 1a). The percentage of phosphorylation stoichiometry can be calculated accordingly. Thus, this motif-targeting kinase reaction offers the key advantages in complexity reduction of the human phosphoproteome (Fig. 1b). Finally, these enzymatically modified phosphopeptides are then purified by IMAC and analyzed by LC-MS/MS. The absolute phosphorylation stoichiometry is decoded by quantifying the heavy/light ratios as shown in Fig. 1a.

### Precision and reproducibility in stoichiometry measurement

**Figure 2:**
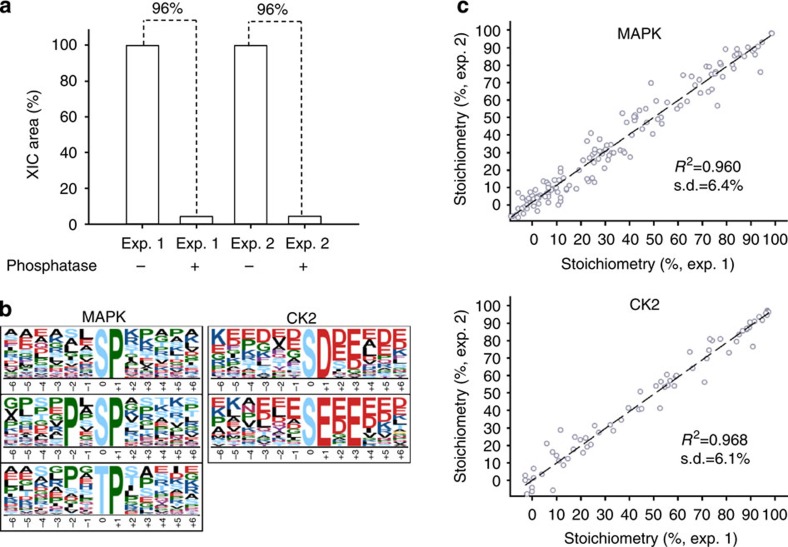
The validity of key steps in the workflow was evaluated by model study of Raji B cells. (**a**) The efficiency of dephosphorylation step was evaluated by quantitative comparison of sum of the extracted ion chromatogram (XIC) from phosphopeptides before and after phosphatase treatment. (**b**) The motif of motif-targeting phosphopeptides after kinase reaction by MAPK and CK2. (**c**) As shown in the scatter plot of the measured stoichiometry between biological duplicate, correlation coefficient between replicate experiments was *R*^2^=0.968 (s.d.=6.1%) for CK2 and *R*^2^=0.960 (s.d.=6.4%) for MAPK, respectively.

The validity of the key steps, including dephosphorylation efficiency, motif-targeting kinase specificity and reproducibility for phosphorylation stoichiometry measurement in the workflow, were evaluated by a model study of Raji B cells (Fig. 2). The efficiency of the dephosphorylation was evaluated by quantitative comparison of the sum of the extracted ion chromatogram from phosphopeptides purified by the first IMAC before and after phosphatase treatment (Fig. 1a). By duplicate analysis, 96% averaged efficiency was obtained for the 1,668 identified phosphopeptides in the mixture of phosphatase-treated and -untreated aliquots (Fig. 2a). For the motif-targeting phosphorylation step, CK2 and MAPK were selected based on the matched motifs of the 2,810 identified phosphorylation sites (Fig. 2b). A total of 1,282 and 541 sites matching to the corresponding phosphorylation motif were commonly phosphorylated in the duplicate CK2 and MAPK reaction, respectively, and the content of the targeting motifs was 80% and 86%, respectively, indicating the high targeting accuracy. As shown in the scatter plot of the measured stoichiometry between biological duplicate (Fig.), correlation coefficient on the stoichiometry measurement between the replicate experiments was *R*^2^=0.968 (s.d.=6.1%) for CK2 reaction and *R*^2^=0.960 (s.d.=6.4%) for MAPK reaction, indicating the good reproducibility in these steps for phosphorylation stoichiometry measurement.

### Phosphorylation stoichiometry profiles in NSCLC

To date, an effective strategy to overcome drug resistance still remains a challenge in the treatment of cancer. In the case of non-small-cell lung cancer (NSCLC), gefitinib, a tyrosine kinase inhibitor (TKI) that targets the epidermal growth factor receptor (EGFR), has been used clinically to treat patients with the EGFR mutation. However, nearly all patients eventually develop resistance to these TKI drugs^10^, which highlighted the need for other approaches to overcome the drug resistance. Although a secondary T790M mutation on EGFR accounts for 50% and c-Met amplification for 20% of the TKI-resistant patients^11^, the resistance mechanisms remain unclear in >30% of TKI-resistant patients who do not have T790M mutations on EGFR^12^. These results highlight the need to search for alternative TKI-resistance signalling pathways or other approaches to identify new therapeutic targets for effective EGFR–TKI treatment^10^.

**Figure 3:**
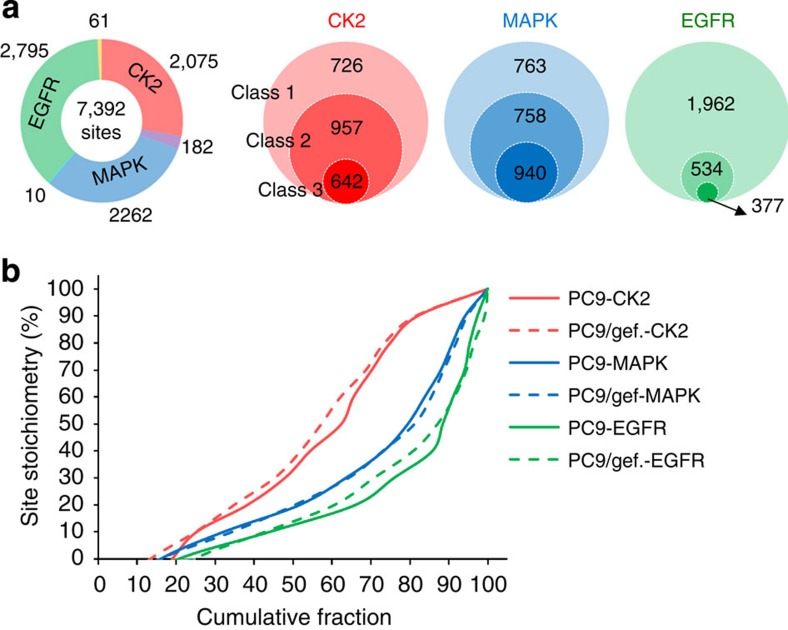
Comparison of phosphorylation stoichiometries between drug sensitive and resistant lung cancer cells. (**a**) The number and specificity of identified phosphorylation sites after kinase reaction (Class 1). The identified phosphorylation sites were further filtered by matching to known motifs (Class 2) and known phosphorylation sites registered in multiple public databases (Class 3). (**b**) Analysis of phosphorylation stoichiometry distribution in motifs matched to EGFR, CK2 and MAPK in drug sensitive (PC9) and resistant (PC9/gef.) lung cancer cells.

Here we apply our motif-targeting approach to measure the absolute stoichiometry for both gefitinib-sensitive cells (PC9) and their gefitinib-resistant counterpart (PC9/gef). PC9 is a gefitinib-sensitive cell line, while PC9/gef is a gefitinib-resistant cell line. Both cell lines harboured an exon 19 deletion mutation on EGFR. The IC_50_ curves of gefitinib to these two lung cancer cell lines were shown in previous study^13^. A total of 2,373 phosphopeptides including 100 multiple phosphorylated peptides were identified in the elution part of 1st IMAC. The 1st IMAC-enriched phosphopeptides from the two cell lines revealed that MAPK and CK2 are the top 2 kinases with upregulated substrates in resistant PC9/gef cells (Supplementary Fig. 1). Therefore, MAPK, CK2 and the known resistant target, EGFR, were employed for motif-targeting phosphorylation. As a result, a total of 7,436 phosphopeptides (7,392 phosphosites) including 276 (3.7%) multiply phosphorylated peptides and 475 (6.4%) phosphopeptides with missed cleavage were identified (Fig. 3a). Phosphorylation of amino acid residues in the vicinity of trypsin cleavage sites has been known to influence the cleavage efficiency, leading to missed cleavage sites in the vicinity of some phosphorylation sites. In our method, we performed the dephosphorylation reaction at protein level instead of peptide level. With nearly complete dephosphorylation efficiency (96%), theoretically, the phosphatase-treated sample as well as the initially unphosphorylated proteins from mocked-treated sample has no remaining phosphorylation to cause missed cleavage. In this date set, up to 14% phosphopeptides identified in the elution part of first IMAC have at least one missed cleavage site. However, only 6.4% motif-targeting phosphorylated peptides have missed cleavage sites. These results indicate that the dephosphorylation at the protein level is useful to improve the efficiency of trypsin and effectively reduce the number of peptides with missed cleavage.

We further applied two filters, matching to known motifs^14^ and matching to known phosphosites registered in multiple public databases^14^,^15^,^16^,^17^,^18^. Finally, 642, 940 and 377 phosphorylation sites from CK2, MAPK, and EGFR, respectively, remained for the subsequent analysis (Fig. 3a). Similar to the current quantitative phosphoproteomic strategies, our method can provide unambiguous quantification for singly phosphorylated peptides. For multiply phosphorylated peptides, the stoichiometry we calculated is the sum of phosphorylated forms on the peptide and could not unambiguously determine the actual phosphorylation stoichiometry of individual sites on multiply phosphorylated peptides.

Figure 3b shows the measured site stoichiometry distribution of the individual cell lines using three kinases. Intriguingly, the phosphorylation sites containing acidic motifs targeted by CK2 generally exhibited higher phosphorylation stoichiometry than sites targeted by MAPK and EGFR both in PC9 and PC9/gef cells. Approximately 30% of CK2 motifs have high stoichiometry (>70%), while <15% of MAPK and EGFR motifs show >70% stoichiometry. In yeast phosphoproteome, Wu *et al*.^7^ reported that high- and low-occupancy was found to be associated with subcellular localization and biological process. To examine the basal level of human phosphoproteome stoichiometry, we categorized the identified human phosphosites into two groups, the medium to high (30–100%) and low (<30%) stoichiometry groups, and performed Gene Ontology enrichment analysis by DAVID. Based on the basal level of our measured phosphoproteome stoichiometry in lung cancer phosphoproteome, interestingly, most proteins with medium to higher stoichiometry (30–100%) were annotated with localization to the nucleus, and a majority of them possessed various functions related to DNA binding, damage and repair (Supplementary Table 1).

To compare the phosphorylation-dependent signalling between PC9 and PC9/gef cells, we analyzed the phosphorylation stoichiometry of these two cells individually. Table 1 shows 13 typical example phosphorylation sites. This strategy can discriminate the quantitative alteration at the stoichiometry level from the alteration at the protein amount. For example, conventional quantitative phosphoproteomics revealed a higher level (ratio=2.4) of pS425 at PTPN3 in the resistant (PC9/gef) cell, whereas our approach shows that such change is due to the increase of stoichiometry (26%) and the protein quantity did not show statistically significant change (protein ratio=1.4). Our motif-targeting approach also demonstrated advantages for capturing low-abundance tyrosine phosphorylation sites, such as HNRNPK (pY280, 20%) and AP2A1 (23%), which were upregulated in phosphorylation degree and not identified by conventional approaches. Compared with previously reported methods^6^, the phosphorylation stoichiometry of a few examples, including SPP1, LCP1, CEBPB and PTPN3, are consistent with the equation developed by Olsen *et al*.^6^ (Supplementary Table 2), but this equation cannot be applied to the remaining four cases in the table owing to its mathematical limitation (see Supplementary Note 1). Among these quantified proteins in Table 1, the amplification of MET has been reported to trigger resistance to EGFR TKIs through the phosphorylation of ERBB3 and activation of the downstream PI3K/Akt pathway^19^. Although the function of pT977 identified in this study in MET remains unknown, the site was confidently identified by this approach (Supplementary Fig. 3a) and phosphorylation was activated from 0% in PC9 cells to 14% in PC9/gef cells.

### Stoichiometry identifies potential resistance targets

**Figure 4:**
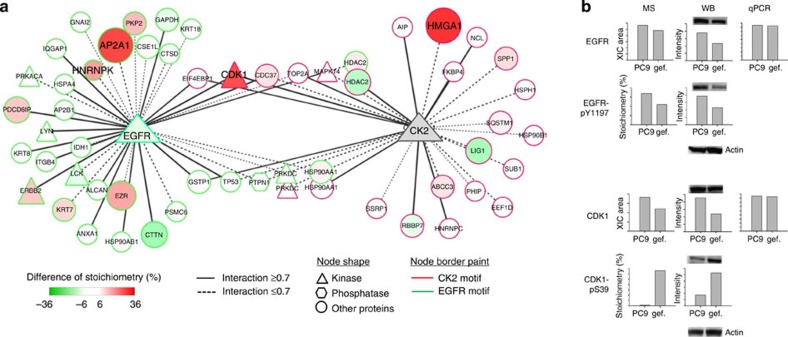
Network analysis of resistance-related elevated phosphorylation in drug sensitive and resistant lung cancer cells. (**a**) Protein network of EGFR and CK2 substrates. Significantly regulated substrates for up and downregulation are shown in red and green, respectively. The size of circle indicates the expression level of phosphorylation stoichiometry. A duplicate protein name means that the different sites were identified within the same protein. (**b**) Validation of the quantitation results of EGFR and CDK1 phosphorylation sites (pY1197 and pS39), protein levels and mRNA levels by mass spectrometry (MS), Western blotting (WB) and real-time RT–PCR (qPCR).

To identify further proteins that might contribute to the different extent of resistance, we constructed a protein–protein interaction network of these identified phosphoproteins with motifs of CK2 and a known oncogenic kinase EGFR. Among their first neighbour protein interaction network (Fig. 4a), the highest phosphorylation alteration (>30% phosphorylation stoichiometry) in resistant PC9/gef was found in three proteins, CDK1, HMGA1 and AP2A1 (localization site information in Supplementary Fig. 3c–e). Interestingly, no changes in either mRNA or protein levels were observed for these proteins (Supplementary Table 3), demonstrating that the resistance-related perturbation of close neighbours in this ‘EGFR‐ and CK2-specific' network may influence only the phosphorylation of their substrates. To verify the accuracy of our approach, the phosphoprotein expression and phosphorylation degree of selected phosphorylation sites with available site-specific antibody, including pY1197 in EGFR and pS39 in CDK1, was validated by Western blotting (Fig. 4b). Based on the good linear correlation (Pearson correlation coefficient of 0.993), the quantitation results of both phosphorylation and protein level from Western blotting and the MS-based quantitation results show good consistency.

Based on the measured absolute stoichiometry, phosphorylation sites of CDK1, HMGA1 and AP2A1 show the highest expression of phosphorylation stoichiometry among these resistance-related elevated phosphorylation sites in the network (Fig. 4a). Besides, S39 in CDK1 has been demonstrated to be a substrate of CK2 (ref. 20) and its function has been known to affect cell cycle regulation^21^. Cell cycle arrest due to DNA damage, known to be caused by many chemotherapeutic drugs, would allow cells to repair damage and confers resistance to anticancer drugs^22^,^23^. For example, a CDK1-dependent pathway has been found to affect how stress hormones mediate drug resistance in human breast cancer cells^24^ In addition, the overexpression of HMGA1 has been reported to enhance oncogenetic miR-222 transcriptional activity in NSCLC^25^ and to promote chemoresistance to gemcitabine in pancreatic cancer cells^26^. In this study, CK2 shows 2.1-fold upregulation in the protein level (Supplementary Fig. 2). The above results suggest that upregulated CK2 may increase the phosphorylation level of its substrate, such as the observed pS39 site in CDK1. CDK1 itself is a highly conserved serine/threonine kinase, and sevenfold higher phosphorylation of its substrate, pT53 in HMGA1, was also observed in resistant PC9/gef. Therefore, the change of phosphorylation stoichiometry may be helpful to characterize the kinase-dependent pathway in drug-resistant lung cancer cells.

## Discussion

To our knowledge, this is the first large-scale approach to assess the phosphorylation stoichiometry of a single-state human proteome. This approach was able to provide reproducible quantitation result (s.d.=±6%). The proof-of-concept experiments on CK2-, MAPK- and EGFR-targeted assays show the advantages of kinase specificity–based complexity reduction of phosphopeptides, which further demonstrates efficient enrichment of low-abundance phosphorylation sites. Such kinase-targeted assays also show high sensitivity for determining the stoichiometry of hundreds of tyrosine phosphorylation sites in 50 μg of cell lysate. In a single cellular state, our results revealed that distinct phosphorylation stoichiometry distributions of different substrates and motifs can be distinguished. Through quantitative measurement of the drug-resistant and sensitive lung cancer cells, this motif-targeting approach suggested potential drug-targeting proteins in the EGFR- and CK2-centred kinase–substrate network. Based on the three most dramatically elevated phosphorylation sites (pT53 in HMGA1, pS39 in CDK1 and pY418 in AP2A1) in response to drug resistance, our stoichiometry measurement also revealed that post-translational phosphorylation changes are much more dramatic than changes either at the protein or the mRNA levels. This approach can provide system-wide maps of protein phosphorylation stoichiometry for either single or multiple cellular states under physiological or pathological regulation.

## Methods

### Chemicals and materials

Protease inhibitor was obtained from Merck (Darmstadt, Germany). Triethylammonium bicarbonate (TEABC), iron (III) chloride (FeCl_3_), acetic acid, formic acid, HPLC-grade acetonitrile (ACN), sodium deoxycholate (SDC), sodium lauroyl sarcosinate (SLS), heavy and light (^13^CD_2_O or ^12^CH_2_O) formaldehyde were purchased from Sigma Aldrich (St Louis, MO, USA). The BCA protein assay kit was obtained from Pierce (Rockford, IL, USA). Trifluoroacetic acid (TFA) and lysyl endopeptidase were purchased from WAKO (Osaka, Japan). Modified sequencing-grade trypsin was purchased from Promega (Madison, WI, USA). Ni-NTA silica resin was purchased from Qiagen (Hilden, Germany). SDB-XC Empore disks were obtained from 3M (St Paul, MN, USA). Water was obtained from a Millipore Milli-Q system (Bedford, MA, USA).

### Cell culture and lysis

The Raji human B cell line was cultured in RPMI-1640 medium (HyClone Logan, UT, USA) supplemented with 10% foetal bovine serum and 1% penicillin G (GibcoBRL, Gaithersburg, MD, USA) at 37 °C in 5% CO_2_. Cells were treated with 500 μM pervanadate (pH 10, with 0.14% H_2_O_2_) for 30 min. After treatment, cells were harvested and washed three times with phosphate-buffered saline and then lysed in lysis buffer (12 mM SDC, 12 mM SLS in 100 mM Tris-HCl, pH 9.0). The protein concentrations were determined via BCA protein assays.

The human lung adenocarcinoma cell line PC9 and derivative PC9/gef clones were gifts from Dr C.H. Yang (Graduate Institute of Oncology, Cancer Research Center, the National Taiwan University). PC9 is a gefitinib-sensitive cell line, while PC9/gef is a gefitinib-resistant cell line. Both cell lines harboured an exon 19 deletion mutation on EGFR. The IC_50_ curve of gefitinib to these two lung cancer cell lines were shown in previous study^13^. Lung cancer cell line (PC9 and PC9/gef.) were grown in RPMI-1640 medium containing 10% FBS and 2 mML-glutamine (all from Life Technologies, Inc.) at 37 °C in a humidified atmosphere of 5% CO_2_ and 95% air. Cells were washed three times with phosphate-buffered saline (0.01 M sodium phosphate, 0.14 M NaCl, pH 7.4) (Sigma, St Louis, MO, USA) and harvested in lysis buffer (12 mM SDC, 12 mM SLS in 100 mM Tris-HCl, pH 9.0). The protein concentrations were determined via BCA protein assays.

### Dephosphorylation and protein digestion

Aliquots of lysates were reduced by 5 mM DTT and alkylated by 2 mM iodoacetamide (IAA) at room temperature for 30 min. The proteins were loaded into 10 kDa molecular weight filter (Amicon Ultra device from Millipore). The device was centrifuged at 14,000 *g* at 25 °C to remove the lysis buffer. Subsequently, the original lysis buffer was replaced by adding 400 μl of 40 mM Tris-HCl (pH=7.5) followed by centrifugation. This step was repeated five times. The resulting concentrate was diluted with 100 μl of 40 mM Tris-HCl (pH=7.5) and separated into two parts. These two parts were treated with or without thermosensitive alkaline phosphatase (TSAP) (protein:TSAP, 100:1, w/w) at 37 °C for 1 h (ref. 27) for dephosphorylation. The reaction was inactivated by heating at 74 °C for 15 min was subjected to proteolytic digestion. The sample solutions were diluted fivefold with 50 mM TEABC and digested with lysyl endopeptidase for 3 h followed by trypsin at 37 °C overnight. The tryptic peptides were acidified by TFA to a final concentration of 0.5%. The resultant peptides were desalted by reversed phase-Stage Tips^28^.

### Stable isotope labelling

Stable isotope labelling was performed as previously described^29^. The tryptic peptides were dissolved in 100 μl 100 mM TEABC. For isotopic dimethyl labelling, the peptides were mixed with 4 μl of 4% ^13^CD_2_O or ^12^CH_2_O, and then 4 μl of freshly prepared 0.6 M sodium cyanoborohydride was immediately added. The mixture was agitated for 60 min at room temperature. The reaction was stopped by adding 16 μl of 1% ammonium hydroxide on ice and agitating the mixture for 1 min. Then dimethyl labelling peptides were mixed with 20 μl of 10% formic acid. The differentially peptides were mixed into one sample and desalted by using reversed phase-Stage Tips^28^.

### Tip-based IMAC

The details can be found from previous reports^30^,^31^. The in-house-constructed IMAC tip was capped at one end with a 20 μm polypropylene frits disk (Agilent, Wilmington, DE, USA) enclosed in a tip-end fitting. The tip was packed with 20 mg of Ni-NTA silica resin. All purification steps for buffer exchange and sample loading involved manipulation via centrifugation. Ni^2+^ ions were removed with 50 mM EDTA in 1 M NaCl. The tip was then activated with 100 mM FeCl_3_ and equilibrated with loading buffer (6% (v/v) acetic acid at pH 3.0) prior to sample loading. Tryptic peptides were reconstituted in loading buffer and loaded onto the IMAC tip. After successive washes with 6% (v/v) acetic acid, 25% ACN and 6% (v/v) acetic acid (AA), the bound peptides were eluted with 200 mM NH_4_H_2_PO_4_. The eluted peptides were desalted using reversed phase-Stage Tips^28^.

### Kinase reaction

The tryptic peptides in the flow through of IMAC were first desalted by using reversed phase-Stage Tips. The desalted peptides were dissolved in kinase reaction buffer (20 mM Tris-HCl, 50 mM KCl and 10 mM MgCl_2_ (pH=7.5) for CK2; 50 mM Tris-HCl, 10 mM MgCl_2_, 0.1 mM EDTA, 2 mM DTT and 0.01% Brij 35 (pH=7.5) for MAPK; 40 mM Tris-HCl, 20 mM MgCl_2_ and 0.1 mg ml^−1^ BSA contacting 50 mM DTT, 2 mM MnCl_2_ (pH=7.5) for EGFR). All recombinant kinases of CK2 and MAPK were obtained from New England BioLabs (NEB, U.K.) except for EGFR (Promega). Each kinase (2500 U, 500 U and 0.2 ug for CK2, MAPK and EGFR, respectively) was added to the desalted peptide mixture containing 1 mM ATP at 30 °C overnight, kinase reactions were stopped by adding TFA to a final concentration of 0.5%. The acidified peptides were desalted by using reversed phase-Stage Tips^28^.

### LC-MS/MS analysis

The TripleTOF 5600 system (AB SCIEX, Concord, ON, Canada) was equipped with a nanoACQUITY UPLC (Waters Corporation, Milford, MA, USA). The 3 μm ReproSil-Pur C18-AQ particles (Dr Maisch, Ammerbuch, Germany) were packed into a 15 cm self-pulled column with a 100 μm inner diameter and 7 μm opening to prepare an analytical column using ‘stone-arch’ frit. The LC system consisted of water with 0.1% FA (buffer A) and ACN with 0.1% FA (buffer B). Peptides were separated through a gradient of up to 80% buffer B over 120 min at flow rate of 500 nl min^−1^. Data were acquired using an ion spray voltage of 2.5 kV, curtain gas at 20 p.s.i., nebulizer gas at 15 p.s.i. and an interface heater temperature of 150 °C. For information-dependent acquisition (IDA), the MS survey scan range was *m*/*z* 300–1,500, and data were acquired for 250 ms. The top 10 precursor ions were selected based on threshold of 100 counts per second in each MS survey scan, and 10 MS/MS scans were performed for 200 ms in each acquisition. The collision energy was automatically adjusted by the rolling CID function of Analyst TF 1.5. To minimize repeated scans, dynamic exclusion was set for 6 s, and the precursor was then removed from the exclusion list.

### Data processing and protein identification

For phosphopeptides identification, the raw MS/MS data were processed using the AB_SCIEX MS Data Converter and analyzed using Mascot (Matrix Science, London, UK; version 2.3) against the SwissPort database (version 57.8, Homo sapiens, 20,329 sequences) with the following constraints: allowing for tryptic peptides with up to two missed cleavage sites, a fragment ion mass tolerance of 0.1 Da and a parent ion tolerance of 20 p.p.m. For unlabelled phosphopeptides, phosphorylation (S, T, Y) and oxidation (M) were selected as variable modifications. For phosphopeptides labelled with dimethyl labelling, dimethyl isotope labels on the peptide N-termini and lysine residues, phosphorylation (S, T, Y) and oxidation (M) were selected as variable modifications. The identification false discovery rate was evaluated by search against a randomized decoy database created by Mascot at the PSM level. In addition, only peptide-spectrum matches (PSMs) with *P* value <0.05 were accepted. The identified phosphopeptides after kinase reaction were classified into three classes. Class 1 means all of identified phosphorylation sites by kinase reaction. Class 2 means the sequence of these phosphopeptides should be corresponded to these kinase motifs in Human Protein Reference Database (HPRD). Class 3 means these phosphorylation sites in Class 2 have been reported in multiple public resource databases^14^,^15^,^16^,^17^,^18^. To evaluate the confidence of phosphorylation site assignment, we report the Mascot Delta Score^32^ in Supplementary Data File 1. According to false localization rate (FLR) calculation for Q-TOF instrument, the 1% and 5% FLR thresholds for the Mascot Delta Score cutoff are 9 and 4, respectively. The raw data sets have been deposited in the ProteomeXchange Consortium (http://proteomecentral.proteomexchange.org) via the PRIDE^33^ partner repository with the data set identifier PXD000728.

### Peptide/protein quantitation

The peptide ion quantitation was performed based on MS1 feature intensity using the DDA quantitation module from DIA-Umpire^34^. The DDA quantitation module accepts the standard mzXML format and starts with background subtraction and centroiding for each MS1 spectrum. The untargeted feature detection algorithm uses a continuous wavelet transformation model on B-spline interpolated peak signals to detect the elution apex and range for each MS1 feature. Isotope peak grouping is then applied to determine *m*/*z* range and to eliminate isolated peaks that are likely to be noise. In this study, detected feature charge states were 2+ to 4+ and the minimum number of isotope peaks was 2. For each identified MS/MS spectrum (false discovery rate<1%), all precursor features observed in MS1 data with close monoisotopic *m*/*z* (within 40 p.p.m.), close retention time (<±1 min) and same charge state were considered as candidates. Among these candidates, the MS1 feature with the closest retention time was determined as the precursor ion for the MS/MS spectrum. The peptide ion intensity and its retention time were determined from the apex of the monoisotopic peak. The quantitation results for phosphopeptides in class 3 were listed in Supplementary Data File 2. In addition, the quantitation result in Fig. 2, Fig. 4c, Table 1, Supplementary Fig. 2, Supplementary Table 2 and Supplementary Table 3 were further manually checked using IDEAL-Q^35^ (data visualization tool).

### Computation analysis

To characterize the amino acid composition of the identified phosphopeptides, the Sequence Logo Generator (http://www.phosphosite.org) was used to visualize significantly conserved sequences. We applied the Motif-X algorithm^8^, to extract phosphorylation motifs. The probability threshold was set at *P*<10^−6^, and the occurrence was set at 20. The extracted phosphorylation motifs were analyzed using the Human Protein Reference Database (http://www.hprd.org/) to search for predicted kinases^14^ and kinase substrates. For the protein–protein interaction analysis, the quantified phosphorylation sites in class 3 which corresponding proteins were searched against the STRING database version 9.1 (ref. 36).

### Western blotting

For Western blotting, cells were lysed by lysis buffer (1% NP-40, 10% glycerol, 150 mM NaCl, 100 mM Sodium phosphate pH 7.2, 1 × EDTA-free protease inhibitor cocktail from Roche) and then incubated on ice for 15 min. Following incubation, the samples were spun at 13,000 *g* for 15 min. The supernatants were collected, and protein concentrations were determined by bicinchoninic acid protein assay kit. For each sample, 50 μg total lysate was separated on 4–12% NuPAGE (Invitrogen). After electrophoresis, the gels were blotted onto PVDF membranes (Millipore) and blocked with blocking buffer (5% BSA in TBS) for 1 h, and then incubated with anti-EGFR antibody (Cell signaling), anti-EGFR phosphosite-specific antibody (Cell signaling), anti-CDK1 antibody (Abnova) and anti-CDK1 (Abgent) phosphosite-specific antibody by 1:1,000 diluted in blocking buffer. After washing with TBST (0.05% Tween-20 in TBS), the membranes were incubated with peroxidase-conjugated second antibodies for developing the signal.

### Real-time RT–PCR

The real-time reverse transcription (RT–PCR) was performed using a standard protocol on an Applied Biosystems 7500 System. Complementary DNA have been purified and diluted to 5 ng μl^−1^. The 20 μl PCR mixture included 2 μl complementary DNA (10 ng), 10 μl 2 × SYBR-green PCR Master Mix (KAPA cat. KK4600), 0.4 μl ROX, 1 μl 4 μM each primer (forward and reverse) (Supplementary Table 4) and 5.6 μl H_2_O. The reactions were incubated in a 96-well plate at 95 °C for 5 min, followed by 40 cycles of 95 °C for 30 s and 60 °C for 45 s. All reactions were run in triplicate. The threshold cycle (CT) is defined as the fractional cycle number at which the fluorescence passes the fixed threshold.

## Author contributions

C.-F.T., Y.I. and Y.-J.C. designed the research. C.-F.T., Y.-T.W., P.-Y.L., H.-Y.Y. and H.-Y.C. participated in the data generation. C.-F.T., C.-C.T. and A.I.N. participated in data quantitation. Y.-J.C., Y.I. and C.-F.T. wrote the manuscript, and Y.-J.C., Y.I., C.-F.T., Y.-T.W. and W.-C.K. edited it.

## Additional information

Accession codes: The raw data sets have been deposited in the ProteomeXchange Consortium (http://proteomecentral.proteomexchange.org) via the PRIDE^33^ partner repository with the data set identifier PXD000728.

**How to cite this article**: Tsai, C.-F. *et al*. Large-scale determination of absolute phosphorylation stoichiometries in human cells by motif-targeting quantitative proteomics. *Nat. Commun.* 6:6622 doi: 10.1038/ncomms7622 (2015).

## Supplementary Material

Supplementary Information Supplementary Figures 1-3, Supplementary Tables 1-4, Supplementary Note 1

Supplementary Data 1 List of total identified phosphopeptides in PC9 and PC9/gef. cells.

Supplementary Data 2 Summary of stoichiometry of motif-targeting phosphopeptides after kinase reaction by CK2, MAPK and EGFR in PC9 and PC9/gef cells.

## Figures and Tables

**Table 1:** Selected examples of differential phosphorylation stoichiometry between drug sensitive (PC9) and resistance (PC9/gef.) lung cancer cell.

**Protein**	**p-site**	**p-site ratio**	**Motif**	**Stoichiometry**			**Protein ratio**
				**PC9**	**PC9/gef.**	**Difference**	
HMGA1	T53	ND	CK2	6%	42%	36%	0.3
AP2A1	Y418	ND	EGFR	11%	34%	23%	1.4
CDK1	S39	ND	CK2	1%	33%	32%	0.7
DKC1	S453	2.4	CK2	45%	77%	32%	2.4
PTPN3	S425	2.4	CK2	27%	56%	29%	1.4
KPNA3	S60	2.5	CK2	34%	58%	24%	3.4
CEBPB	T235	2.3	MAPK	41%	62%	21%	1.7
HNRNPK	Y280	ND	EGFR	0%	20%	20%	0.9
MET	T977	ND	MAPK	0%	14%	14%	1.7
SF3A1	S359	3.5	CK2	100%	100%	0%	2
NOP56	S520	5.1	CK2	63%	61%	−2%	2.1
EGFR	Y1197	ND	EGFR	34%	22%	−12%	0.8
HDAC2	S394	0.1	CK2	100%	86%	−14%	1.7

‘ND’ means that this phosphorylation site was not identified by conventional phosphoproteomic strategy.
