# Supplementary material for: Large-scale determination of absolute phosphorylation stoichiometries in human cells by motif-targeting quantitative proteomics
Source: Nat Commun. 2015 Mar 27;6:6622. doi: 10.1038/ncomms7622 (PMC4389224; doi:10.1038/ncomms7622)
Supplement: Supplementary Information — Supplementary Figures 1-3, Supplementary Tables 1-4, Supplementary Note 1 [file ncomms7622-s1.pdf]

**Supplementary Figure 1**

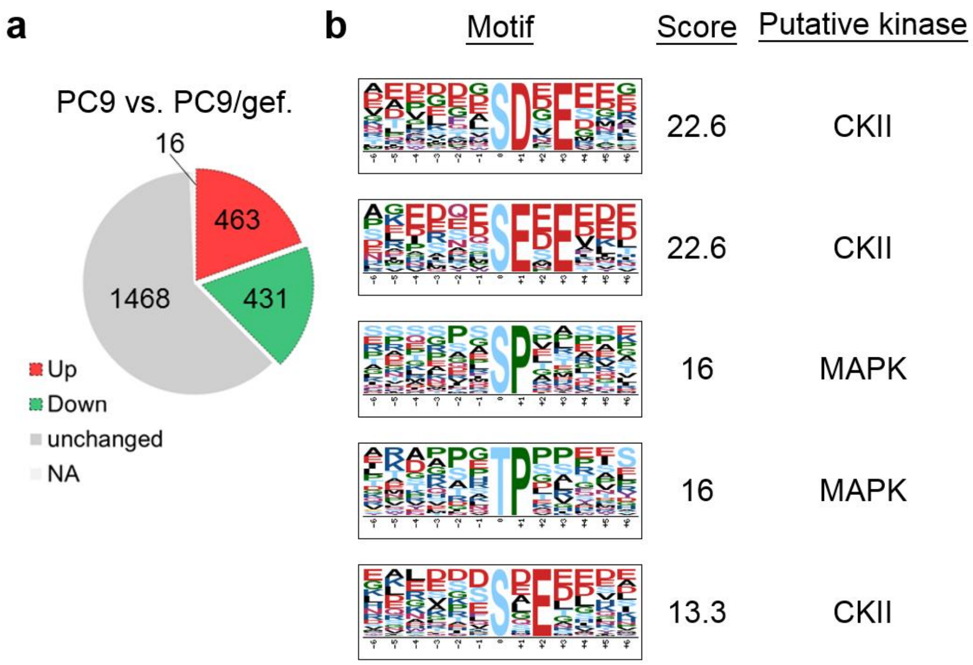


Supplementary Figure 1. The quantitation result of PC9/gef. versus PC9 using a conventional quantitative phosphoproteomic strategy. (a) The pie chart shows the relative percentage of increase or decrease in the levels of 2378 identified phosphopeptides in drug-sensitive PC9 cells compared to drug-resistant PC9/gef. (b) Five sequence motifs were enriched from 463 up-regulated phosphopeptides by motif-X analysis; 2 motifs were matched to CK2 and represented the top ranking.

**Supplementary Figure 2**

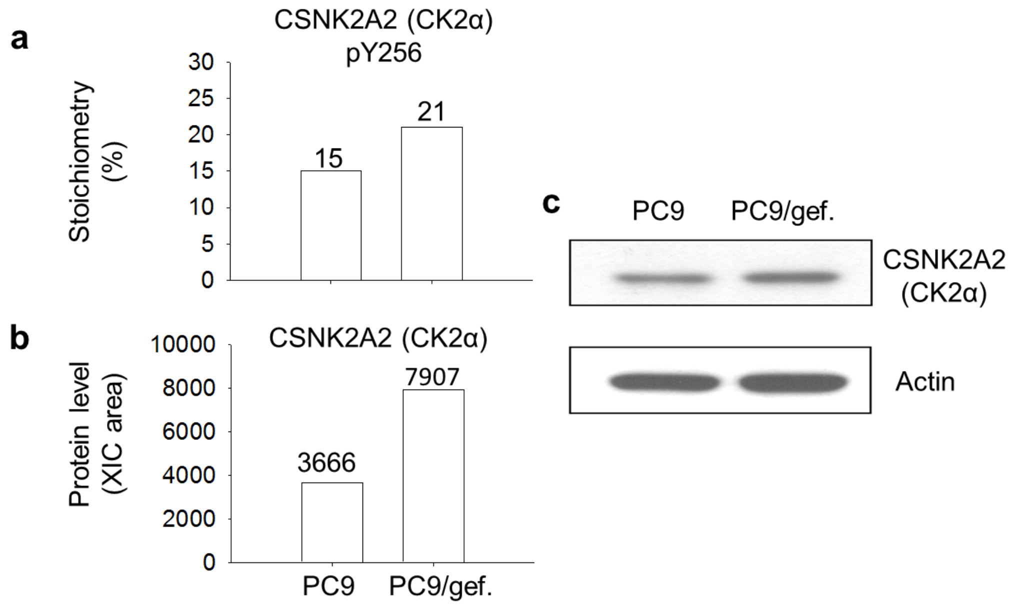


Supplementary Figure 2. Comparison of differential protein expression and phosphorylation level of CK2α in PC9 and PC9/gef cells. (a) Calculated phosphorylation stoichiometry of pY256 in CK2α in PC9 (15%) and PC9/gef (21%). No significant difference in phosphorylation stoichiometry was observed between these two cell lines. (b) Our approach also measured the protein level of CK2α. The extracted ion chromatogram (XIC) areas of CK2α in PC9 and PC9/gef showed approximately 2-fold up-regulation in PC9/gef. (c) The quantitation result at protein level was also confirmed by western blot analysis.

## Supplementary Figure 3

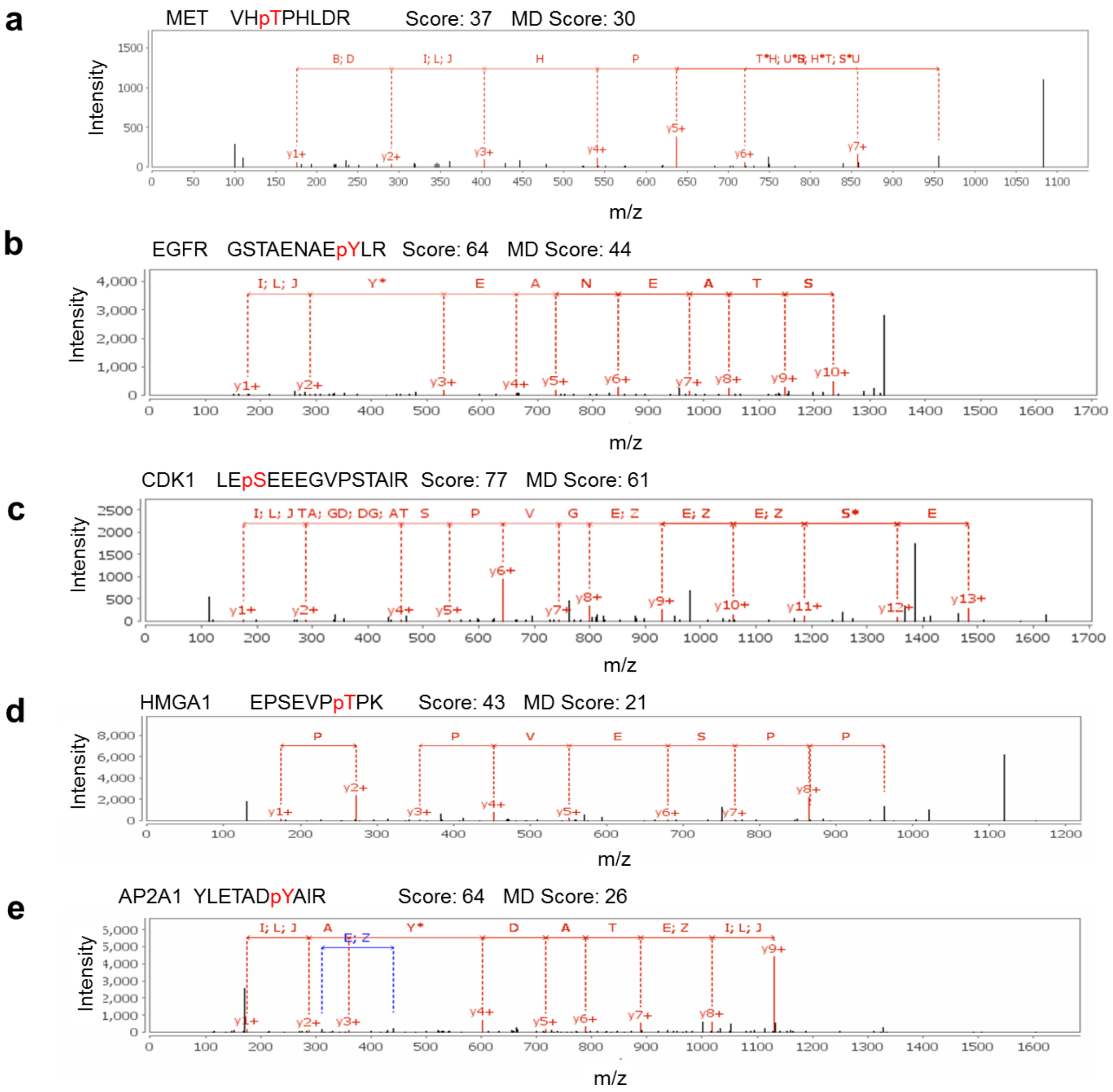


Supplementary Figure 3. The MS/MS spectra and MD scores of identified phosphorylation sites in (a) MET (pY977), (b) EGFR (pY1197), (c) CDK1 (pS39), (d) HMGA1 (pT53), and (e) AP2A1 (pY418).

**Supplementary Table 1**

**a** <10%

| Term | PValue |
|---|---|
| acetylation | 6.19E-06 |
| oxidoreductase | 7.94E-05 |
| nadp | 3.42E-03 |
| host-virus interaction | 6.08E-03 |
| blocked amino end | 9.33E-03 |
| actin binding | 1.13E-02 |
| multifunctional enzyme | 2.34E-02 |
| Isomerase | 2.96E-02 |
| endoplasmic reticulum | 3.39E-02 |
| methylation | 3.64E-02 |
| signal | 4.23E-02 |
| cytoplasm | 4.31E-02 |
| disulfide bond | 4.80E-02 |
| mRNA transport | 4.80E-02 |

**b** 0~30%

| Term | PValue |
|---|---|
| Secreted | 6.19E-03 |
| calmodulin-binding | 1.25E-02 |
| Chaperone | 1.64E-02 |
| cytoskeleton | 2.02E-02 |
| disease mutation | 2.34E-02 |
| host-virus interaction | 2.46E-02 |
| duplication | 2.79E-02 |
| cell membrane | 4.46E-02 |
| actin binding | 4.78E-02 |

**c** 30~75%

| Term | PValue |
|---|---|
| alternative splicing | 2.07E-03 |
| nucleus | 2.22E-03 |
| phosphoprotein | 2.54E-03 |
| dna-binding | 7.08E-03 |
| DNA damage | 9.89E-03 |
| chromosomal protein | 1.86E-02 |
| metal-binding | 4.10E-02 |
| bromodomain | 4.25E-02 |
| actin-binding | 4.28E-02 |
| cytoskeleton | 4.58E-02 |
| dna repair | 4.74E-02 |

**d** >75%

| Term | PValue |
|---|---|
| phosphoprotein | 4.14E-08 |
| nucleus | 6.12E-05 |
| zinc-finger | 6.89E-05 |
| alternative splicing | 3.24E-04 |
| zinc | 6.76E-04 |
| transcription regulation | 8.90E-04 |
| Transcription | 9.97E-04 |
| metal-binding | 3.27E-03 |
| polymorphism | 1.77E-02 |
| dna-binding | 2.27E-02 |

Supplementary Table 1. Protein function annotation for different groups of phosphorylation stoichiometry in PC9 cells. These identified phosphoproteins were categorized into two groups, the medium to high (c group: 30%-75%; d group: 75-100%) and low (a group: <10%; b group: 10-30%) stoichiometry groups. Gene Ontology enrichment analysis was performed by DAVID. The green, red and blue colors indicate the annotated protein functions related to the nucleus, cytosol and membrane, respectively.

**Supplementary Table 2**

| Protein | Phosphorylation sites | Phosphorylation stoichiometry (%) | | | |
|---|---|---|---|---|---|
| | | This study | | Other equation[5] | |
| | | PC9 | PC9/gef. | PC9 | PC9/gef. |
| SPP1 | S215 | 10 | 20 | 1 | 12 |
| LCP1 | S7 | -1 | 2 | 1 | 3 |
| NOP56 | S520 | 63 | 61 | Nan | Nan |
| DKC1 | S453 | 45 | 77 | Nan | Nan |
| KPNA3 | S60 | 34 | 58 | Nan | Nan |
| CEBPB | T235 | 41 | 62 | 52 | 70 |
| SF3A1 | S359 | 100 | 100 | Nan | Nan |
| PTPN3 | S425 | 27 | 56 | 35 | 61 |

Nan: the stoichiometry can't be calculated according to the equation[5]

Supplementary Table 2. Selected examples to compare the results of the phosphorylation stoichiometry measurement by this study and the mathematical equation established by Olsen et al.[3]

**Supplementary Table 3**

| | QPCR | | | Protein level | | | Stoichiometry (%) | | | |
|---|---|---|---|---|---|---|---|---|---|---|
| | PC9 | PC9/gef. | Ratio | PC9 | PC9/gef. | Ratio | Site | PC9 | PC9/gef. | Difference |
| EGFR | 17.0 | 16.8 | 0.99 | 1142 | 963 | 0.84 | Y1197 | 34 | 22 | -12 |
| CSNK2A2 | 16.4 | 16.3 | 0.99 | 3666 | 7907 | 2.16 | Y256 | 15 | 21 | 6 |
| CDK1 | 17.8 | 16.9 | 0.95 | 7309 | 4821 | 0.70 | S39 | 1 | 33 | 32 |
| HMGA1 | 17.2 | 17.3 | 1.01 | 720 | 219 | 0.30 | T53 | 6 | 42 | 36 |
| AP2A1 | 21.4 | 21.0 | 0.98 | 3949 | 5353 | 1.40 | Y418 | 1 | 34 | 33 |
| HNRNPK | 19.4 | 19.2 | 0.99 | 286 | 261 | 0.90 | Y280 | 0 | 20 | 20 |
| MET | 17.3 | 17.9 | 1.03 | 409 | 713 | 1.70 | Y977 | -3 | 14 | 17 |

Supplementary Table 3. Comparison of the differential expression levels in mRNA, protein and phosphorylation stoichiometry in selected examples of phosphoproteins. Examples were selected from the network shown in Figure 2: EGFR (pY1197), CSNK2A2 (pY256), CDK1 (pS39), HMGA1 (pT53), AP2A1 (pY418), HNRNPK (pY280) and MET (pY977).

**Supplementary Table 4**

| Gene | | Primer sequence |
|---|---|---|
| *AP2A1* | Forward | 5' CAACAAGGAACTGGCCAACAT 3' |
| | Reverse | 5' GGCCAAGCAGGAAGATGAAA 3' |
| *HMGA1* | Forward | 5' AGCGAAGTGCCAACACCTAAG 3' |
| | Reverse | 5' CTCCTCTTCCTCCTTCTCCAGTTT 3' |
| *HNRNPK* | Forward | 5' GGTGGTAGCAGAGCTCGGAAT 3' |
| | Reverse | 5' AACCAACCATGCCGTCGTAA 3' |
| *MET* | Forward | 5' GCTTTGCAGCGCGTTGA 3' |
| | Reverse | 5' CCTGATCGAGAAACCACAACCT 3' |
| *EGFR* | Forward | 5' GACGCAGATAGTCGCCCAAA 3' |
| | Reverse | 5' ACGGTAGAAGTTGGAGTCTGTAGGA 3' |
| *CSNK2A2* | Forward | 5' TGTTCGCATTGCCAAGGTT 3' |
| | Reverse | 5' TGAATGTTGTCCCAGGATATCG 3' |
| *CDK1* | Forward | 5' CCTAGCATCCCATGTCAAAAACTT 3' |
| | Reverse | 5' CAGTGCCATTTTGCCAGAAA 3' |
| *TBP* | Forward | 5' CTGGCCCATAGTGATCTTTGC 3' |
| | Reverse | 5' TCAATTCCTTGGGTTATCTTCACA 3' |

Supplementary Table 4. Design of primer (forward and reverse) of selected cDNA.

**Supplementary Note 1**

Proportion (a) Proportion (b)

$$= \frac{N_L{}^{phos}}{N_L{}^{nonphos}} \qquad = \frac{N_H{}^{phos}}{N_H{}^{nonphos}}$$

$$= \frac{1-\frac{y}{x}}{\frac{x}{z}-1} \qquad = \frac{x}{y}\left(\frac{1-\frac{y}{x}}{\frac{x}{z}-1}\right)$$

x : ratio of modified peptides

y : ratio of unmodified peptides

z : ratio of protein

*Olsen et al. Sci. Signal.* **3**, ra3 (2010).

Occupancy of cell state 1: a/(1+a) Occupancy of cell state 2: b/(1+b)

We have tested different assumption. There are four conditions that will fail in the calculation.

1. "Stoichiometry unchanged and Protein level unchanged" which means the ratio of modified peptides (x) and protein (z) will be 1. Therefore, the denominator will be 0 and the phosphorylation stoichiometry can't be calculated
2. "Stoichiometry unchanged and Protein level Up" which means the ratio of modifiedpeptides (x) and protein (z) will be the same. Therefore, the x/z will be equal to 1. According to the equation, the denominator will be 0 and the phosphorylation stoichiometry can't be calculated
3. "100 % Stoichiometry" which means the ratio of unmodified peptides (y) will be 0. Therefore, the denominator will be 0 and the phosphorylation stoichiometry can't be calculated
4. "0 % Stoichiometry" which means the ratio of modified peptides (x) can't be calculated. Therefore, the phosphorylation stoichiometry can't be calculated

Supplementary Note 1. The limitation of phosphorylation occupancy calculation equation which developed by Olsen et al. (Sci. Signal. 3, ra3, 2010)
